# Static Low-Angle Squatting Reduces the Intra-Articular Inflammatory Cytokines and Improves the Performance of Patients with Knee Osteoarthritis

**DOI:** 10.1155/2019/9617923

**Published:** 2019-10-30

**Authors:** Zhihong Zhao, Rui Wang, Yu Guo, Lianxu Chen, Kai Wang, Haibo Zhou, Hongwei Li, Jingbin Zhou

**Affiliations:** ^1^Department of Orthopedics, Beijing Second Hospital, Beijing 100031, China; ^2^Department of Sports Biomechanics, College of Sports Human Science, Beijing Sport University, Beijing 100084, China; ^3^Department of Orthopedics, Beijing Tsinghua Changgung Hospital, Beijing 100044, China; ^4^National Institute of Sports Medicine, Beijing 100061, China; ^5^Orthpedic Department, General Hospital of PLA, Beijing 100853, China

## Abstract

Osteoarthritis (OA) is one of the major diseases leading to disability, and inflammation plays an important role in the pathogenesis of OA. However, inflammation of OA is multifactorial, chronic, and in low intensity, which makes drug-based immunotherapy difficult. Here, we have designed a novel method of exercise—static low angle squat (SLAS), which reduces the intra-articular inflammation of OA knee as well as strengthens the vastus medialis of quadriceps. A two-year follow-up trial of current exercise methods demonstrated long-term, significant improvement in pain relief, range of motion, muscle strength, and knee stability.

## 1. Introduction

Osteoarthritis (OA) of the knee that is characterized by focal loss of articular cartilage and marginal bone formation, resulting in joint space narrowing and osteophytosis, is one of the top causes of disability among adults [1]. About 12% of Americans above the age of 60 experience symptomatic knee OA [2]. In China, the incidence is 49% of the retired population in the city, and 38% of the people above 60 years in the country side [3].

Although the etiology of OA is incompletely understood, aging, damage of the articular cartilage, and the relevant inflammation process clearly play a major role. After the cartilage is damaged, the degraded metabolites in the joint cavity leads to synovitis. The inflammatory cytokines, proteases, and prostaglandins are therefore secreted into the joint cavity, altering the physicochemical properties of the synovial fluid. The cartilage that rubs abnormally are susceptible to further damage [4]. In recent years, there is growing evidence that inflammation, which is mainly mediated by the innate immune system, plays a key role in the OA pathogenesis. However, there is so far no clinical evidence that patients with knee osteoarthritis could benefit from the immunotherapy, including the corticosteroid injections [5]. This might be due to the fact that the pathogenesis of OA involves multiple inflammatory factors that are often present in a chronic state, relatively low grade.

Researchers showed that patients can benefit from strength training, which has positive effects on pain scores and functional outcomes in knee OA [6]. More importantly, long-term exercise could reduce the inflammatory condition of knee osteoarthritis. Clinical applications show that both failure to remain active and disuse of the affected limb can accelerate impaired joint mechanics and potentially result in articular cartilage softening and matrix dysfunction, leading to more rapid cartilage degeneration [7]. Exercise strengthening the overlying muscles and soft tissue structures of joint could improve gait mechanics, joint movement, fascial tension, and tissue circulation [8]. These improvement can further upregulate costimulatory factor PGC-1α in skeletal muscle, which negatively regulates NF-*κ*B and inhibits the enhancement of IL-1*β*, IL-6, and TNF*α*-induced by NF-*κ*B [9]. These cytokines have been shown to be involved in the regulation of cartilage homeostasis, by promoting cartilage catabolism and inhibiting anabolism.

New evidence has emerged suggesting that moderate physical activity may be beneficial to the people with osteoarthritis [10]. However, there is always discussion about the exercise safety and a more beneficial, long-term, nonpharmacology approach for the rehabilitation of OA is needed. Squat is one of the most frequently used exercises in the field of strength and conditioning, which recruits most of the lower body musculature, including the quadriceps femoris, hip extensors, hip adductors, hip abductors, and triceps surae. In addition, significant isometric activity is conferred to a wide range of supporting muscles to facilitate postural stabilization of the trunk, of which over 200 muscles are activated during squat performance [11]. Nevertheless, squats can be performed at a variety of depths, and deep squat, especially with weight bear, can be harmful to the knee [12]. In China, a static squat that is performed in the low angle, called “horse stance (Ma Bu),” has been put into practice for a long history, which provides a reliable and safe isometric exercise strengthening not only quadriceps but also a set of surrounding muscles. In this study, we designed a novel approach of static low angle squat (SLAS), based on the traditional Chinese exercise. We found that, after one year of SLAS exercise, the levels of proinflammatory factors, TNF*α* and IL-1*β*, were significantly reduced in the synovial fluid of OA knee, while the anti-inflammatory cytokine IL-10 increased. Moreover, we performed a large scale of comparative trials to evaluate this method in the OA populations and observed a significant improvement of the HSS scores in the treatment group, including both the functional recovery and pain reduction. Our results suggest that SLAS exercise provides a reliable approach in the rehabilitation of OA, which could be readily accepted by OA patients for long-term benefit.

## 2. Methods

### 2.1. Subjects

The inclusion criteria are as follows: the patient was between 54 and 65 years of age, unilaterally or bilaterally involved, with pain in and around the knee joint. Subjects were excluded if they had any deformity of the knee, hip, or back, or had any central or peripheral nervous system involvement, or had received steroids or intra-articular injection within the previous three months or received physiotherapy treatment in the past 6 months.

The study was approved by our Institutional Ethical Committee (IEC), and written consent was obtained from all the participants.

### 2.2. Methods

Subjects in the experiment underwent 2 years of exercise (*n* = 55). Exercise twice a day for 30 minutes each time. Patients who cannot be completed in one go can perform multiple exercises and adjust the interval accordingly.

  SLAS exercise: patients stand with the legs apart; the distance between the knees as well as the feet should be as wide as the shoulders, and then they try to squat down. When bending the knees, patients should try to keep the back straight and adjust the angle of knees from straight down as close to 90 degrees as possible but no less than 90 degrees. However, it should not reach the position that patients feel painful so that any potential damage should be avoided.

Synovial fluids were taken from the OA knee before the exercise and 12 month after. Red blood cells were removed by centrifugation, and the supernatants were frozen at −80°C. Cytokine and BMP-7 (bone morphogenetic proteins) levels were determined with ELISA (R&D) according to the manufacturer's protocol.

The outcome measures for this study were HSS scores, including pain, knee function, ROM, quadriceps strength, deformity, and stability. These variables were measured on both sides of the legs, respectively. All the measurements were taken at baseline (January 2013), 1 year (December 2013), and 2 years (December 2014) after exercise.

### 2.3. Statistical Analysis

Statistical analyses were performed using the Statistical Package of Social Science (SPSS software version 18.0). The means and standard deviations were computed, and the one sample paired *t*-test was used to compare pre- and postintervention measures on the pain, knee function, ROM, quadriceps strength, deformity, and stability, respectively. The level of statistical significance was set at *p* < 0.05.

## 3. Results

To strengthen the quadriceps and the surrounding ligament, we designed a static squat protocol, SLAS, based on the horse stance (Ma Bu) that is the traditional exercise in China. The patients flex their knees (not less than 90 degrees) while exercising and keep their back straight. The knee angle may be reduced after long-term exercise, but the reduction must be the extent to which the patient does not feel pain.

The synovial fluid of OA knee was taken before the exercise and 12 months after the exercise. Samples were analyzed for TNF*α*, IL-1*β,* and IL-10 (Table 1). The concentration of proinflammatory cytokine TNF*α* and IL-1*β* showed significant decreases after the SLAS exercise compared to before (*p* < 0.001). In contrast, a highly significant increase was found for the anti-inflammatory cytokine IL-10 (*p* < 0.001), suggesting that the SLAS exercise could reduce the inflammation in the OA knee. Correspondingly, the level of BMP-7 in the synovial fluid is significantly reduced in the patients 12 months after SLAS exercise (*p* < 0.001) (Table 1). BMP-7 is an important bone conversion biomarker, which has been shown to correlate with the disease severity of knee OA.

The subjects in the experimental performed the exercise for 24 months. Hospital for Special Surgery (HSS) scores [13] were used to evaluate the outcome for this study. The control and exercise group were chosen randomly, and the patients showed similar HSS scores before the follow-up trial (Table 2). Pain and functional scores were significantly improved after 12 months and 24 months of exercise (*p* < 0.001). After 12 months of regular exercise, there was also a significant difference in ROM and muscle strength scores (*p* < 0.001), with a slight increase in the second year of exercise (*p* < 0.05). The deformity scores were significantly enhanced in the first year (*p* < 0.01) but not altered in the second year. However, there was no significant difference in knee stability between the scores before and after the exercise program (*p*=0.319) (Tables 3 and 4).

OA often causes joint space narrow that mostly takes place on the inner board of the knee, due to the bias of leg structure and the barycenter of our bodyweight. SLAS exercise could strengthen particularly the vastus medialis muscle of quadriceps and increase the joint space. Consistent to this hypothesis, our follow-up trial demonstrated a significant pain relief and the restoring of functions in the OA patients.

## 4. Discussion

In this study, we designed a novel exercise approach, SLAS, which has a broad effect on the muscle on the back and surrounding knees. Our study indicated that SLAS exercise reduces the inflammation condition of OA knee. With the large scale of comparative trials, we found that SLAS could in long term reduce the pain and improve the function, mobility, and stability of knee, providing a reliable method for the rehabilitation of OA patients.

The pathogenesis of OA begins with cartilage damage and matrix protein release and is exacerbated by the triggering of DAMP (danger associated molecular patterns) signaling and subsequent chronic inflammation. However, single factor immunotherapy has proven to be useless for OA treatment because of the involvement of multiple inflammatory factors. Furthermore, the low inflammatory intensity in OA does not meet the principles of conventional anti-inflammatory therapies. Therefore, OA treatment requires long-term, reliable treatment for low-grade chronic inflammation. Our data suggest that low-dose long-term exercise such as SLAS can reduce the inflammatory state of the OA knee joint, which may be achieved not only by strengthening the muscles but also by soothing the microenvironment niche of innate immunity in the joint space.

Strength of the quadriceps is one of the intrinsic factors that has been shown to affect the knee joint functions and is closely associated with disability [14]. Many of the resistance-training protocols have focused on strengthening quadriceps, of which straight leg raising (SLR) exercise is often considered. With patient sitting and raising tibias, SLR could largely improve the muscle of quadriceps; however, such exercises may neglect the practice of hamstring that is critical for the stability of knees. It is known that quadriceps strengthening alone is not sufficient to treat the subgroup patients of OA such as tibiofemoral OA [15]. SLAS may be able to motivate the muscle not only on the leg but also in the hip and back, providing more comprehensive improvement of knee stability. Therefore, there is need for studying the effect of SLAS on the strengthening of hamstring or hip abductor as compared to quadriceps-only programs.

Rehabilitation of OA is usually focused on symptom management, where pain relief, improved joint function, and joint stability are the main goals of treatment. SLAS exercise provides a new rehabilitation program without the risk of causing further damage, which improves knee stability, strengthens the medial femoral muscle of the quadriceps, and expands the joint space. Also, our two-year follow-up trial showed that SLAS exercise could counteract muscle atrophy, reduce pain, and partially restore knee function. In addition, SLAS is designed based on the posture of Chinese traditional exercise, the action and concept of which would be readily accepted by a large population. In practice, SLAS can be performed at home and adjusted accordingly by the patient, which is feasible in the long run.

## Figures and Tables

**Table 1:** Cytokine levels in the synovial fluid of OA knee.

	TNF*α* (pg/ml)	IL-1*β* (pg/ml)	IL-10 (pg/ml)	BMP-7 (pg/ml)
Before	22.43 ± 4.31	80.23 ± 6.54	45.14 ± 5.36	10.50 ± 2.54
After (12 months)	14.07 ± 2.89^*∗*^	43.75 ± 5.23^*∗*^	90.45 ± 4.53^*∗*^	3.27 ± 1.38^*∗*^

“^∗^Significant difference.”

**Table 2:** Prescores for HSS.

Group	HSS score	Each item scoring					
		Pain	Function	ROM	Muscle strength	Flexion deformity	Knee stability
Control	61.30 ± 6.40	16.33 ± 2.22	7.95 ± 1.84	12.00 ± 2.31	8.04 ± 1.45	7.76 ± 0.74	9.21 ± 1.28
Exercise	61.44 ± 6.56	16.19 ± 2.43	7.93 ± 1.92	12.00 ± 2.33	7.99 ± 1.62	7.72 ± 0.78	9.17 ± 1.34

**Table 3:** 12 months postexercise scores for HSS (CI 0.95).

Group	HSS score	Each item scoring					
		Pain	Function	ROM	Muscle strength	Flexion deformity	Knee stability
Control	62.04 ± 6.44	16.19 ± 2.52	7.93 ± 1.80	12.00 ± 2.27	7.99 ± 1.19	7.72 ± 0.72	9.17 ± 1.17
Exercise	75.35 ± 9.00^*∗*^	22.23 ± 4.85^*∗*^	9.89 ± 2.74^*∗*^	14.40 ± 2.12^*∗*^	9.62 ± 1.16^*∗*^	9.72 ± 0.78^*∗*^	9.46 ± 1.09

**Table 4:** 24 months postexercise scores for HSS (CI 0.95).

Group	HSS score	Each item scoring					
		Pain	Function	ROM	Muscle strength	Flexion deformity	Knee stability
Control	60.94 ± 9.56	16.85 ± 5.04	7.84 ± 2.77	12.36 ± 2.12	7.64 ± 1.15	7.76 ± 0.74	9.46 ± 1.09
Exercise	85.77 ± 7.50^*∗*^	24.01 ± 4.11^*∗*^	18.94 ± 2.71^*∗*^	14.20 ± 2.27^*∗*^	9.43 ± 1.25^*∗*^	9.76 ± 0.74^*∗*^	9.41 ± 1.23

## Data Availability

The data used to support the findings of this study are available from the corresponding author upon request.
